# Evaluation of multiple measures of antiretroviral adherence in the Eastern European country of Georgia

**DOI:** 10.7448/IAS.17.1.18885

**Published:** 2014-04-09

**Authors:** Nikoloz Chkhartishvili, Nino Rukhadze, Mariam Svanidze, Lali Sharvadze, Jack A Dehovitz, Tengiz Tsertsvadze, Louise-Anne McNutt, Carlos del Rio

**Affiliations:** 1 Infectious Diseases, AIDS and Clinical Immunology Research Center, Tbilisi, Georgia; 2 Faculty of Medicine, Tbilisi State University, Tbilisi, Georgia; 3 Department of Medicine, SUNY Downstate Medical Center, Brooklyn, NY, USA; 4 Department of Epidemiology and Biostatistics, University at Albany School of Public Health, Rensselaer, NY, USA; 5 Hubert Department of Global Health, Emory University Rollins School of Public Health, Atlanta, GA, USA

**Keywords:** antiretroviral therapy, adherence, Eastern Europe, injection drug use, viral suppression

## Abstract

**Introduction:**

There is little information on adherence to antiretroviral therapy (ART) in the Eastern European region. This prospective study evaluated multiple measures of adherence and their association with viral suppression among HIV patients in Georgia.

**Methods:**

A prospective cohort study enrolled 100 consecutive antiretroviral-naïve adult (age ≥18 years) patients, who were followed for three months. Adherence was assessed by medication refill and three self-report measures (an AIDS Clinical Trial Group [ACTG] tool for four-day adherence, a visual analogue scale [VAS] and a rating task for 30-day adherence). The VAS represented a line anchored by 0 and 100% corresponding to the percentage of prescribed doses taken. The rating task asked patients to rate their ability to take all medications as prescribed, with responses categorized into six levels of adherence: very poor (0%), poor (20%), fair (40%), good (60%), very good (80%) and excellent (100%). Patients with adherence of ≥95% by medication refill, ACTG and VAS, and ≥80% by rating task, were defined as adherent.

**Results:**

Of 100 patients enrolled, eight had missing data and were excluded from analysis. Among the remaining 92 patients, the median age was 39 years, and 70% were men. Major modes of HIV acquisition were injection drug use (IDU; 47.3%) and heterosexual contact (44.1%). The proportions of adherent patients were as follows: 68% by medication refill, 90% by ACTG questionnaire, 38% by VAS and 42% by rating task. On average, four months after commencing ART, 52 (56.5%) patients had a viral load <400 copies/ml and 26 (28.3%) patients had a viral load <50 copies/ml. Of 43 persons with a history of IDU, 22 (51.2%) reached a viral load of <400 copies/ml. In multivariate analysis, only refill adherence was a statistically significant predictor of viral suppression of <400 copies/ml: the risk ratio was 1.7 (95% CI: 1.1–2.8). Refill adherence, VAS and rating task were associated with viral suppression of <50 copies/ml. Non-IDUs were twice as likely to achieve viral load <50 copies/ml compared to IDUs. Refill adherence had the largest area under the receiver-operating characteristic curve for predicting viral suppression.

**Conclusions:**

Medication refill adherence was the strongest predictor of viral suppression. IDUs can achieve optimal virologic outcomes, but may require additional adherence support.

## Introduction

Access to antiretroviral therapy (ART) is rapidly expanding worldwide [1]. People in resource-limited countries who are HIV-positive can now enjoy the benefits of ART, which were previously limited to those in developed, resource-rich nations [2,3]. Given the central role of adherence in the success of the treatment, the expansion of ART programmes in low- and middle-income countries created the need for indicators to assess programme performance with respect to adherence [4]. Incomplete adherence leads to treatment failure, the development of drug resistance, disease progression and death [5–8]. However, the absence of a “gold standard” in adherence makes measurement challenging [9].

Adherence assessment methods commonly used in clinical trials and observational studies include electronic drug monitoring, prescription (medication) refills, pill counts and various self-reported methods. Electronic monitoring and unannounced pill counts were shown to be the best objective measures for real-time assessment of adherence [10]. However, their use in clinical practice is limited because of high costs and logistical issues [11]. Medication refill is another objective measure of adherence, and it is calculated from routinely available information in pharmacy or drug-dispensing records. Its use is feasible in variety of settings and has been validated in both developed and developing countries [12,13]. The self-report remains the most frequently used adherence measure because of its low cost and simplicity. Although the accuracy of self-reports has been questioned because of social desirability and recall bias, several approaches have shown good correlation with objective adherence measures and viral suppression [14–17].

Formerly part of the Soviet Union, Georgia is an independent nation situated in the Caucasus region. Similar to other former Soviet Union (FSU) countries, the HIV epidemic in Georgia is closely tied to injection drug users (IDUs), who account for 54% of all reported HIV infections. As of 31 December 2012, a cumulative 3642 cases of HIV infection were reported in Georgia. Antiretroviral drugs have been available in the country since the 1990s, but the access was limited only to those able to afford them. However, beginning in 2004, through support from the Global Fund, Georgia became the only FSU country to initiate universal access to ART [18]. IDU has been identified as a risk factor for poor adherence, and many national programmes and clinicians elect not to initiate ART in patients who are active IDUs [19,20]. Therefore, better adherence monitoring is necessary for achieving optimal treatment outcomes in countries where the HIV epidemic is primarily driven by IDUs. This prospective study evaluated multiple measures of adherence and their association with viral suppression among HIV patients living in Georgia.

## Methods

### Study design

The prospective cohort study was conducted at the Infectious Diseases, AIDS and Clinical Immunology Research Center (IDACIRC) in Tbilisi, Georgia, which is the country's referral institution for HIV diagnosis, treatment and care. The study included 100 consecutive antiretroviral-naïve adult (age ≥18 years) patients first initiating ART at the IDACIRC during the period from June 2010 to December 2010. Patients were approached after the first refill of their ART prescription at IDACIRC. Consenting patients were followed for three months after enrolment. The protocol included an enrolment interview and three follow-up adherence assessments at monthly intervals during drug refill visits. The main outcome measures were adherence to ART assessed by four different methods, and viral suppression was defined as plasma HIV-1 RNA levels (viral load) of <400 copies/ml and <50 copies/ml.

### ART programme

The National ART Program is coordinated by the IDACIRC. HIV-related treatment and care services are provided at the IDACIRC clinic in the capital city of Tbilisi as well as at three affiliated regional centres in the cities of Kutaisi, Batumi and Zugdidi. All persons living with HIV in Georgia are referred to and followed by this centre and its affiliates.

Provision of therapy is governed by the National HIV/AIDS Treatment and Care guidelines, which were developed based on the guidelines of the World Health Organization (WHO) European Region. At the time the study was conducted, ART was indicated in all patients with CD4 <200 cells/mm^3^ as well as those with hepatitis B or C and tuberculosis (TB) regardless of CD4 count.

The recommended initial regimen consists of two nucleoside reverse transcriptase inhibitors (NRTIs) and one non-nucleoside reverse transcriptase inhibitor (NNRTI). A ritonavir (r)-boosted protease inhibitor (PI) is recommended in cases when an NNRTI cannot be prescribed. As per the Georgian national guidelines, the standard of ART monitoring relies upon laboratory monitoring of CD4 count, HIV-1 viral load and resistance assessment based on resistance genotype detection when indicated.

Antiretroviral drugs are dispensed at four centres at monthly intervals. Monthly drug pick-up encounters serve as important points for adherence assessment by self-report, and refill adherence using pharmacy records is used to monitor drug pick-up practices. Patients demonstrating suboptimal adherence (such as late drug pick-up and virological non-response) receive additional home-based adherence support by mobile units operating countrywide.

### Adherence measures

Adherence was treated as a continuous variable expressed as a percentage. Adherence was assessed by medication refill and three self-report measures. Provisions were made to maximize honest responses, including selecting interviewers who were not directly involved in ART provision and ensuring the confidentiality of data.

*Medication refill*: Medication (or prescription) refill adherence was the objective measure of adherence used in the study. Adherence was defined as the number of days for which the supply of medication was dispensed divided by the days between prescription fills [21]. If the supply of medication exceeded the number of days between refills, then adherence was truncated to 100%.

*Self-report measures* assessed adherence over either a four-day or 30-day period prior to the study visit.

*Four days*: AIDS Clinical Trials Group (ACTG) adherence was used to assess four-day adherence. The questionnaire queried the patient on the number of doses missed of an ARV drug over each of the four days prior to the study visit. We calculated adherence as 1 – (number of doses missed for the day/number of doses prescribed), expressed as a percentage [22].

*Thirty days*: One-month adherence was assessed using two methods. (1) The *visual analogue scale* (VAS) is represented with a line anchored by 0 and 100%, corresponding to the percentage of prescribed doses taken. Patients were asked to put a mark on that line [23]. (2) The *rating task* asked patients a specific question – “Please rate your ability to take all your medications as prescribed” – which had six predefined response categories: very poor, poor, fair, good, very good and excellent. For the purposes of analysis, each response category was assigned a corresponding adherence score: very poor – 0%, poor – 20%, fair – 40%, good – 60%, very good – 80% and excellent – 100% [16].

### Other measures

Information on sociodemographic characteristics, substance abuse and the presence of symptoms of depression was collected at the study entry. Patients were screened for illicit drug use and alcohol abuse using the Drug Abuse Screening Test (DAST-10) and the Alcohol Use Disorders Identification Test (AUDIT), respectively [24,25].

Medical records were reviewed for CD4 cell count, viral load and co-infection with hepatitis B virus (HBV), hepatitis C virus (HCV), TB, and other comorbidities. All laboratory assays were performed at the laboratory service of the IDACIRC. CD4 cell count and viral load were measured at physicians’ discretion as part of the standard of care; this is usually performed every four months after stating ART. Therefore, measurements closest to the enrolment and last study visits were abstracted.

Information on mode of HIV transmission was also abstracted from the medical records. Mode of transmission in Georgia is classified based on a presumed risk hierarchy, taking into account the efficiency of HIV transmission associated with the reported behaviour or exposure to the virus. It is assigned by an experienced counsellor during post-test counselling after confirmation of HIV infection.

### Statistical analysis

Distributions of study variables were assessed in bivariate and multivariate analyses. Comparisons were tested using two-sample or paired *t*-tests as appropriate. The correlation between continuous variables was tested by Spearman's correlation coefficient. The ability of each adherence measure to predict viral suppression was evaluated by calculating the area under the receiver-operating characteristic (ROC) curve.

Associations between multiple covariates and viral suppression were tested in a modified Poisson regression model with robust variance estimates [26]. For this analysis, the primary outcome measure was defined as achievement of an undetectable viral load. Patients were categorized as adherent if they had taken at least 95% of the dispensed dosage as measured by medication refill, four-day ACTG questionnaire and VAS, or 80% of the dispensed dosage by the rating task. A purposeful variable selection strategy was used to fit the multivariate regression models [27]. The initial model included variables significant at *p*≤0.10 in bivariate analysis. In the next steps of variable selection, covariates were retained in the model if they were significant at the *p*≤0.10 level or showed a confounding effect evaluated as a >10% change in parameter estimates. After reducing the full model, variables not initially included were added back one at a time and evaluated as referenced above together with previously selected significant variables and confounders. Statistical analyses were performed using SAS 9.2 (SAS Institute, Cary, NC, USA).

### Ethical approval

The study was reviewed and approved by the Institutional Review Boards (IRBs) of the IDACIRC and State University of New York Downstate Medical Center. Patients were approached right after they picked up the first prescription of antiretroviral drugs at the IDACIRC clinic. Patients were detailed about the study verbally and given the opportunity to read the informed consent form; they also were provided sufficient time for questions. After ensuring that patients comprehended the study-related information, voluntary agreement to participate was documented by signing the informed consent form. Patients agreed to participate in three interviews over the three-month period and have their medical records reviewed for study purposes.

## Results

One hundred patients were enrolled in the study. Eight had missed study visits, and because of missing data on adherence as well as other follow-up data, these patients were excluded from analysis. Of the excluded patients, five patients had transferred to a regional clinic, and three missed study visits. Of these three patients lost to follow-up, one was an active drug user and another had a history of IDU.

Table 1 summarizes the baseline characteristics of the 92 patients included in the analysis. Their median age was 39 years, and 70% were men. One third had college or university level of education, and approximately half were unemployed. Major modes of transmission were IDU (47.3%) and heterosexual contact (44.1%). Ongoing substance abuse measured by the DAST tool demonstrated that 16.1% of patients had a moderate to severe level of drug abuse, including 10.8% of participants with signs of harmful or hazardous alcohol use as assessed by the AUDIT tool. Patients were started on ART on average 14 months after registering at the IDACIRC. Thirty-one percent of patients had AIDS-defining conditions, including 19 (20.4%) patients with active tuberculosis and 11 (11.8%) patients with *Pneumocystis jiroveci* pneumonia (PCP). Nearly half of the patients were co-infected with HCV, and two patients had evidence of chronic HBV infection. The median CD4 cell count at treatment initiation was 136 cells/mm^3^ (interquartile range [IQR]: 55–194), and the median viral load was 5.7 log_10_ copies/ml (IQR: 5.2–6.1). The majority of patients were started on an NNRTI-based regimen (88%), and the most commonly prescribed NRTI backbone was zidovudine (AZT)+lamivudine (3TC).

**Table 1:** Baseline characteristics

Characteristic	*n*=92
Age, mean years (*SD*)	39 (8.0)
Gender, *n* (%)	
Male	64 (70.0)
Female	28 (30.0)
Marital status, *n* (%)	
Married	53 (57.6)
Not married	39 (42.4)
College or university, *n* (%)	29 (31.5)
Unemployed, *n* (%)	51 (55.4)
Mode of HIV transmission, *n* (%)	
Injection drug use	43 (46.7)
Heterosexual contact	41 (44.6)
Male-to-male sex	8 (8.7)
Moderate to severe level of drug abuse (DAST), *n* (%)*	14 (15.2)
Harmful or hazardous alcohol use (AUDIT), *n* (%)**	10 (10.9)
Viral load, median log_10_ copies/ml (IQR)	5.7 (5.2–6.2)
CD4 cell count, median cells/mm^3^ (IQR)	136 (55–194)
Comorbidities, *n* (%)	
HCV	44 (47.8)
HBV	2 (2.2)
Tuberculosis	19 (20.7)
PCP	11 (12.0)
Other	9 (9.8)
ART regimen, *n* (%)	
AZT+3TC+EFV	35 (38.0)
ABC+3TC+EFV	23 (25.0)
TDF+FTC+EFV	16 (17.4)
AZT+3TC+NVP	5 (5.4)
TDF+FTC+NVP	2 (2.2)
AZT+3TC+LPV/r	3 (3.2)
ABC+3TC+LPV/r	4 (4.4)
TDF+FTC+LPV/r	4 (4.4)
Time to ART start after HIV diagnosis, mean months (*SD*)	14 (27)

* Drug Abuse Screening Test (DAST) score ≥3

** Alcohol Use Disorders Identification Test (AUDIT) score ≥8.

Mean adherence as measured at each monthly assessment did not vary significantly over the study period. Therefore, the third-month adherence measurements were used in analyses. At the end of follow-up, the mean medication refill adherence was 94%. For self-reported measures, the mean adherence was 97% by four-day ACTG questionnaire, 89% by VAS and 67% by rating task. The proportion of patients showing ≥95% adherence was 68% by medication refill, 90% by four-day ACTG questionnaire and 38% by VAS. Overall, 85, 91 and 74% of patients showed adherence of at least 90% by medication refill, four-day ACTG questionnaire and VAS, respectively. Forty-two percent of patients showed ≥80% adherence by rating task (Table 2).

**Table 2:** Comparison of adherence by gender and substance abuse

		Gender			History of IDU			Active IDU*			Active alcohol use**		
		
	Total (*n*=92)	Male (*n*=64)	Female (*n*=28)	*p*	Yes (*n*=43)	No (*n*=49)	*p*	Yes (*n*=14)	No (*n*=78)	*p*	Yes (*n*=10)	No (*n*=82)	*p*
Refill adherence													
Mean (%)	94	94	95	0.54	94	95	0.44	95	95	0.99	88	95	0.11
>95% adherent, *n* (%)	63 (68.5)	40 (62.5)	23 (82.1)	0.06	25 (58.1)	38 (77.6)	0.05	6 (42.9)	57 (73.1)	0.03	3 (30.0)	39 (70.9)	0.01
>90% adherent, *n* (%)	78 (84.8)	54 (84.4)	24 (85.7)	0.87	36 (83.7)	42 (85.7)	0.79	12 (85.7)	66 (84.6)	1.0	7 (70.0)	71 (86.6)	0.18
4-day ACTG													
Mean (%)	97	96	100	0.04	94	100	0.03	91	98	0.04	85	98	0.21
>95% adherent, *n* (%)	83 (90.2)	56 (87.5)	27 (96.4)	0.27	35 (81.4)	48 (98.0)	0.01	9 (64.3)	74 (94.9)	0.004	7 (70.0)	76 (92.7)	0.06
>90% adherent, *n* (%)	84 (91.3)	56 (87.5)	28 (100.0)	0.10	35 (81.4)	49 (100)	0.002	9 (64.3)	75 (96.2)	0.002	7 (70.0)	99 (93.9)	0.04
VAS													
Mean (%)	89	88	92	0.01	87	91	0.05	86	90	0.20	74	91	0.05
>95% adherent, *n* (%)	35 (38.0)	20 (31.3)	15 (53.6)	0.04	12 (27.9)	23 (46.9)	0.06	2 (14.3)	33 (42.3)	0.07	0 (0.0)	35 (42.7)	0.01
>90% adherent, *n* (%)	68 (73.9)	43 (67.2)	25 (89.3)	0.04	27 (62.8)	41 (83.7)	0.02	8 (57.1)	60 (76.9)	0.18	4 (40.0)	64 (78.1)	0.02
Rating													
Mean (%)	67	66	71	0.07	65	69	0.07	61	68	0.06	50	69	0.009
>80% adherent, *n* (%)	39 (42.4)	23 (35.9)	16 (57.1)	0.06	14 (32.6)	25 (51.0)	0.07	2 (14.3)	37 (47.4)	0.04	0 (0.0)	39 (47.6)	0.004

* Drug Abuse Screening Test (DAST) score ≥3

** Alcohol Use Disorders Identification Test (AUDIT) score ≥8.

Response distribution to self-reported measures was as follows: 83 (90.2%) patients reported perfect (100%) adherence by four-day ACTG questionnaire; the most common response to rating task was “good” (48 [52.2%] patients), and none reported perfect adherence; and the most frequent response to VAS was 90% – for 33 (35.9%) patients – and only 6 (6.5%) patients rated their adherence as 100%.

The mean medication refill adherence was similar when compared with sociodemographic characteristics, history of substance abuse or comorbidities. Exploring differences in the percentage of adherent patients showed that a greater proportion of females and non-substance abusers were ≥95% adherent by medication refill. There were no differences in the proportions of adherent patients when medication refill adherence was dichotomized at the 90% level (Table 2). Females and persons without a history of substance abuse were also more adherent by self-report instruments. The differences remained statistically significant regardless of the adherence definition used except for the impact of active IDU on VAS adherence (Table 2). Adherence did not differ when stratified by other sociodemographic characteristics, comorbidities and prescribed ART regimen.

The association between adherence and plasma HIV viral load was also evaluated. The viral load was measured on average four (IQR: 3–5) months after commencing ART, and 11 (11.9%) patients had their viral load measured at least six months after initiating therapy. The mean viral load drop from baseline was 2.9 log_10_ copies/ml (*p*<0.0001). Fifty-two (56.5%) patients had a viral load <400 copies/ml, and 26 (28.3%) patients had a viral load <50 copies/ml. Of 43 persons with a history of IDU, 22 (51.2%) reached a viral load of <400 copies/ml and six (13.9) completely suppressed a viral load below 50 copies/ml. There was a negative correlation between adherence and viral load. Correlations were significant for all studied measures (refill adherence: −0.38, *p*=0.0002; four-day ACTG: −0.29, *p*=0.005; VAS: −0.41, *p*<0. 0001; and rating: −0.30, *p*=0.004).

**Figure 1:**
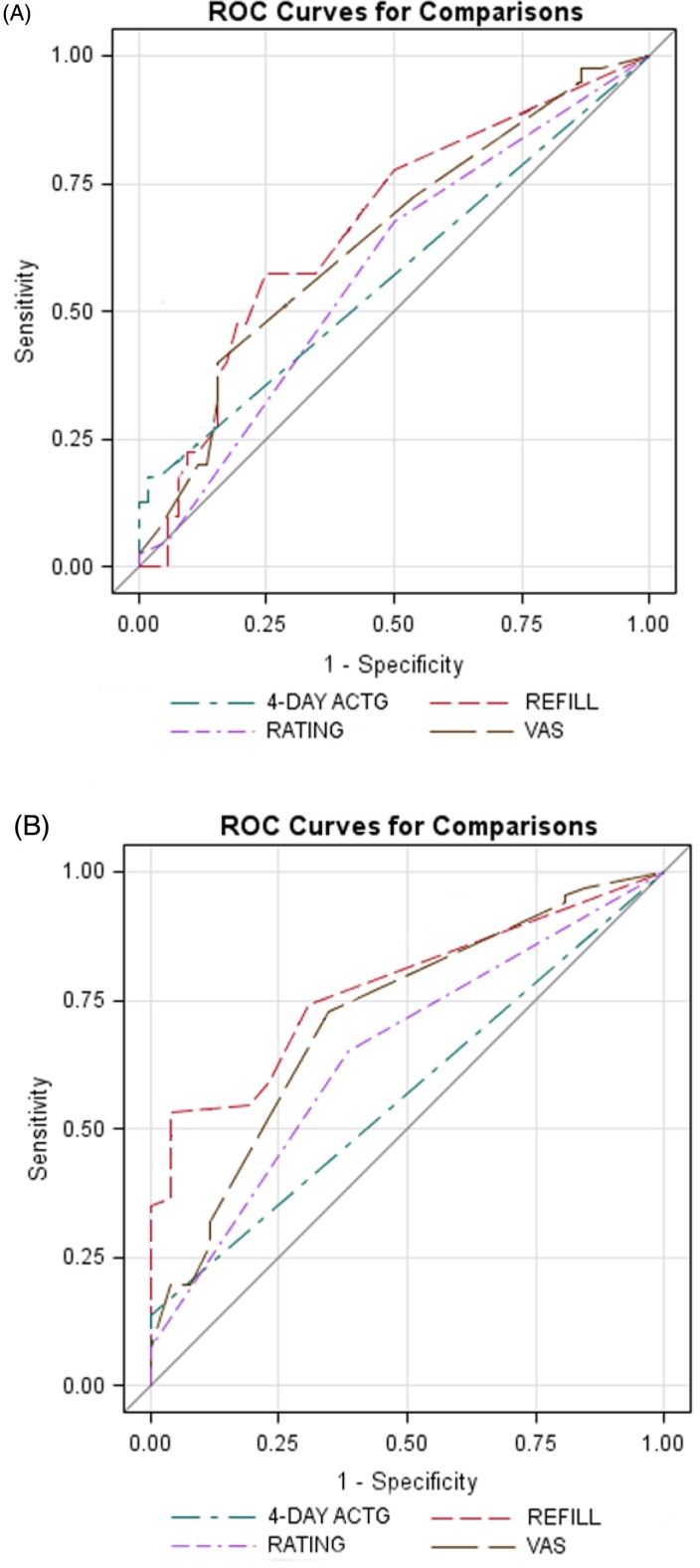
Receiver-operating characteristic (ROC) curves for predicting viral suppression. A. ROC curve for viral suppression <400 copies/ml. Area under the curve for each adherence measure: refill adherence – 0.67 (95% CI: 0.56–0.78); four-day ACTG questionnaire – 0.57 (95% CI: 0.51–0.64); VAS – 0.64 (95% CI: 0.53–0.75); and rating task – 0.58 (0.48–0.69). B. ROC curve for viral suppression <50 copies/ml. Area under the curve for each adherence measure: refill adherence – 0.77 (95% CI: 0.68–0.86); four-day ACTG questionnaire – 0.57 (95% CI: 0.53–0.61); VAS – 0.72 (95% CI: 0.60–0.83); and rating task – 0.65 (0.54–0.76).

The area under the ROC curves for predicting undetectable viral load for both levels of suppression (<400 copies/ml and <50 copies/ml) was larger for refill adherence compared to other measures (Figure 1). However, at a suppression level of <400 copies/ml, the difference between the areas under the curves for studied measures was not statistically significant. At a suppression level of <50 copies/ml, the area under the curve for refill adherence was significantly larger compared to that of four-day ACTG (*p*<0.0001) and rating task (*p*=0.04), but not compared to the area for VAS (*p*=0.43). There was also a statistically significant difference between areas generated by VAS and four-day ACTG (*p*=0.01). No difference was found between four-day ACTG and rating task.

Multivariate Poisson regression models with robust variance estimates were fitted to evaluate predictors for each level of viral suppression. Of four adherence measures, only refill adherence was a statistically significant predictor of viral suppression of <400 copies/ml (risk ratio [RR]: 1.7; 95% CI: 1.1–2.8, *p*=0.04). Other significant predictors of viral suppression were a baseline viral load <100,000 copies/ml and an AZT-based regimen. Active IDUs showed a confounding effect only in the four-day ACTG model (Table 3). Neither gender nor substance abuse showed significant associations.

**Table 3:** Predictors of viral suppression <400 copies/ml

		Undetectable viral load (<400 c/ml)					
		
			Bivariate	Model 1: refill	Model 2: ACTG	Model 3: VAS	Model 4: rating
			
	Total *N*	*n* (%)	RR (95% CI, *p*)	RR (95% CI, *p*)	RR (95% CI, *p*)	RR (95% CI, *p*)	RR (95% CI, *p*)
Gender							
Female	28	18 (64.3)	1.2 (0.8–1.7, 0.32)				
Male	64	34 (53.1)					
Mode of transmission							
Non-IDU	49	30 (61.2)	1.2 (0.8–1.7, 0.33)				
IDU	43	22 (51.2)					
Active IDU*							
No	78	47 (60.3)	1.7 (0.8–3.5, 0.09)	1.5 (0.7–3.1, 0.29)	1.4 (0.7–2.6, 0.36)	1.6 (0.8–3.3, 0.19)	1.5 (0.7–3.2, 0.27)
Yes	14	5 (35.7)					
Active alcohol use**							
No	82	48 (58.5)	1.5 (0.7–3.2, 0.32)				
Yes	10	4 (40.0)					
Baseline viral load							
<100,000	16	13 (81.3)	1.6 (1.1–2.2, 0.05)	1.6 (1.2–2.1, 0.003)	1.7 (1.2–2.3, 0.001)	1.6 (1.2–2.1, 0.003)	1.6 (1.2–2.1, 0.003)
>100,000	76	39 (51.3)					
Time to viral load measurement after ART start (per month increase)	92		1.1 (1.0–1.2, 0.004)	1.1 (1.0–1.2, 0.003)	1.1 (1.0–1.2, 0.001)	1.1 (1.1–1.2, 0.0006)	1.1 (1.1.–1.2, 0.0003)
NRTI component							
AZT-based	43	29 (67.4)	1.4 (1.0–2.1, 0.05)	1.4 (1.0–1.9, 0.04)	1.4 (1.0–2.0, 0.04)	1.4 (1.0–2.0, 0.04)	1.5 (1.02,1, 0.03)
TDF or ABC based	49	23 (46.9)					
Refill adherence							
>95%	63	42 (66.7)	1.9 (1.1–3.3, 0.04)	1.7 (1.1–2.8, 0.04)			
<95%	29	10 (34.5)					
4-Day ACTG							
>95%	83	50 (60.2)	2.7 (0.8–9.3, 0.04)		2.5 (0.7–8.3, 0.14)		
<95%	9	2 (22.2)					
VAS							
>95%	35	24 (68.6)	1.4 (1.0–2.0, 0.07)			1.3 (0.9–1.8, 0.10)	
<95%	57	28 (49.1)					
Rating							
>80%	39	26 (66.7)	1.4 (1.0–1.9, 0.09)				1.4 (1.0–2.0, 0.07)
<80%	53	26 (49.1)					

* Drug Abuse Screening Test (DAST) score ≥3

** Alcohol Use Disorders Identification Test (AUDIT) score ≥8.

Predictors of complete viral suppression (<50 copies/ml) were assessed for refill adherence, VAS and rating task (Table 4). None of those reporting less than 95% adherence by the four-day ACTG questionnaire completely suppressed the virus. Consequently, the association could not be measured. In the multivariate model, all measures were significantly associated with undetectable viral load: refill adherence had an RR of 9.2 (1.5–56.3, *p*=0.02); VAS, an RR of 2.9 (1.6–5.2, *p*=0.0009); and rating, an RR of 2.4 (1.2–4.7, *p*=0.009). Histories of IDU and AZT-based ART regimens were significantly associated with the outcome in all three models. Non-IDUs were more than twice as likely to achieve complete viral suppression compared to IDUs. Although gender was significantly associated with the outcome in bivariate analysis, it has lost significance in multivariate analysis, likely due to correlation with substance abuse variables.

**Table 4:** Predictors of viral suppression <50 copies/ml

		Undetectable viral load (<50 c/ml)					
		
			Bivariate	Model 1: refill	Model 2: VAS	Model 3: rating
			
	Total *N*	*n* (%)	RR (95% CI, *p*)	RR (95% CI, *p*)	RR (95% CI, *p*)	RR (95% CI, *p*)
Gender						
Female	28	13 (46.4)	2.3 (1.2–4.3, 0.01)			
Male	64	13 (20.3)				
Mode of transmission						
Non-IDU	49	20 (40.8)	2.9 (1.3–6.6, 0.004)	2.1 (1.0–4.4, 0.04)	2.4 (1.2–4.9, 0.02)	2.2 (0.9–5.1, 0.07)
IDU	43	6 (13.9)				
Active IDU*						
No	78	25 (32.1)	4.5 (0.7–30.5, 0.10)			
Yes	14	1 (7.1)				
Active alcohol use**						
No	82	25 (30.5)	3.0 (0.5–20.1, 0.27)			
Yes	10	1 (10.0)				
Baseline viral load						
<100,000	16	9 (56.3)	2.5 (1.4–4.6, 0.006)	1.9 (1.1–3.5, 0.02)	1.7 (1.0–3.0, 0.07)	1.9 (1.0–3.6, 0.05)
>100,000	76	17 (22.4)				
Time to viral load measurement after ART start (per month increase)	92		1.1 (0.9–1.3, 0.14)	1.1 (0.9–1.3, 0.13)	1.2 (1.0–1.4, 0.03)	1.2 (1.0–1.5, 0.02)
NRTI component						
AZT based	43	17 (39.5)	2.2 (1.1–4.3, 0.02)	2.1 (1.1–3.8, 0.02)	2.4 (1.3–4.4, 0.005)	2.4 (1.2–4.7, 0.007)
TDF or ABC based	49	9 (18.4)				
Refill adherence						
>95%	63	25 (39.7)	11.5 (1.6–80.9, <0.0001)	9.2 (1.5–56.3, 0.02)		
<95%	29	1 (3.5)				
VAS						
>95%	35	17 (48.6)	3.1 (1.5–6.1, 0.0007)		2.9 (1.6–5.2, 0.0009)	
<95%	57	9 (15.8)				
Rating						
>80%	39	16 (41.0)	2.2 (1.1–4.3, 0.02)			2.4 (1.2–4.7, 0.009)
<80%	53	10 (18.9)				
Four-day ACTG						
>95%	83	26 (31.3)				
<95%	9	0 (0.0)				

* Drug Abuse Screening Test (DAST) score ≥3

** Alcohol Use Disorders Identification Test (AUDIT) score ≥8.

## Discussion

In this study, we evaluated the performance of four measures of adherence, including a medication refill–based objective measure and three self-reported methods. The mean medication refill adherence was 94%, and 68% of patients demonstrated adherence of at least 95%, which is comparable to adherence reported elsewhere [28]. Moreover, at the 90% cut-off, medication refill adherence compares favourably to levels seen in both industrialized and resource-limited countries [29].

Responses to self-reported measures varied substantially. Findings of our study are consistent with previous reports, indicating that multi-item self-report methods with shorter recall periods (≤7 days) tend to overestimate actual adherence [15,16,30–32]. In our study, 90% of patients reported perfect (100%) adherence on the four-day ACTG, which is significantly higher than that for time periods covered by refilled medication dosages.

Similar to the findings in other studies, substance abuse was associated with lower levels of adherence [33–36]. Lowest rates were observed among both active drug and alcohol users. However, because the study assessed few active substance abusers, it is difficult to make meaningful comparisons. Noteworthy, a difference by medication refill was observed only at the ≥95% adherence cut-off, while for self-reported measures persons with substance abuse had lower adherence regardless of the definition of adherence. Overall, 58 and 84% of patients with a history of IDU had a drug supply for 95 and 90% of the time, respectively, as measured by medication refill data. These levels are in the range of adherence reported in other studies involving non-IDU populations as well [28,29].

In our cohort, women showed better adherence than men by all measures, except for the four-day ACTG model. This could be explained by the fact that none of the women had a history of substance abuse and the difference diminished after adjusting for IDU. This finding underlines the role that substance use plays in observed gender-specific differences [37].

Our study design allowed us to evaluate adherence measures in relation to virological outcome. All adherence measures correlated with viral suppression. ROC statistics for predicting viral suppression at both the <400 and <50 copies/ml levels were larger for refill adherence, followed by VAS. Refill adherence and VAS yielded significantly larger areas under the curve compared to four-day ACTG and rating task at a viral suppression level of <50 copies/ml. In multivariate analysis, refill adherence was the strongest predictor of viral suppression, and it was the only adherence measure significantly associated with achievement of a viral load <400 copies/ml.

Other factors associated with viral suppression included a lower baseline viral load (<100,000 copies/ml) and the use of a zidovudine (AZT)-containing regimen. While the influence of baseline viral load is well described, the association found between the use of an AZT-containing regimen and viral suppression should be interpreted with caution. Regimens were selected by treating physicians based on patient characteristics and clinical conditions. Consequently, the observed difference could have resulted from these and other unmeasured confounding factors, and may not represent a real difference.

Because of the small number of active substance abusers, the ability to evaluate associations with the outcome was limited. Patients with and without a history of IDU had similar rates of viral suppression at levels <400 copies/ml, but non-IDUs were more likely to achieve viral load levels <50 copies/ml. Overall, these findings suggest that undetectable viral load is achievable among IDUs, but this population may require additional adherence support to achieve complete viral suppression.

Our study is subject to several limitations. We used medication refill data as an objective measure of adherence, which estimates maximum possible adherence without accounting for the possibility that patients may not take all refilled medications. Therefore, refill adherence is also prone to bias, but it is more accurate than self-reported methods. This study included ART-naïve patients who were followed for the first three months of therapy. In addition, the majority of patients had advanced diseases and such patients might be more motivated, demonstrating better adherence early in the course of ART. High early adherence may wane over time, influencing virologic outcomes as well [5,38]. However, studies indicate that adherence remains stable over time [39] and also suggests a critical role of early adherence for long-term virological success [40].

Another limitation is the small sample size, which limits the generalizability of our findings and also negatively influenced statistical power to assess associations in certain population subgroups, such as active substance abusers. In addition, of 100 initially enrolled patients, eight were excluded from analyses. Because of the small number of patients with missing data, we decided to exclude them rather than impute missing data. Also, the dramatic differences in the proportions of patients showing >95% adherence (68% refill adherence, 90% four-day ACTG and 38% VAS), as well as no 100% adherence by rating task, raise questions regarding whether patients had fully understood the self-reported measures. Interviewers were appropriately trained on how to instruct patients and correctly administer tools; however, the possibility of misunderstanding by patients cannot be ruled out.

## Conclusions

Our study has important clinical and public health implications. This study confirmed that medication refill adherence is a useful tool for predicting virological outcomes and can be used for monitoring adherence at both patient and programme levels [12,13,41]. Because of the above-described disadvantages of refill adherence, self-report remains an important tool for evaluating adherence in routine clinical practice. Based on our study, VAS seems to be an appropriate measure in Georgian settings. It did not have a significant ceiling effect and showed an association with viral suppression. In addition, VAS is simple to administer and can identify patients at risk of virologic failure, who can benefit from adherence discussion and counselling [42,43].

To the best of our knowledge, this is the first prospective evaluation of multiple adherence measures reported from countries of the FSU. This region has the fastest growing HIV epidemic among IDUs and the lowest ART coverage [44]. With the exception of Georgia, all other countries of the region provide less than half of eligible patients with ART [1]. This low ART coverage results in part from structural barriers for IDUs to access the life-saving therapy [45,46]. Addressing these barriers and providing a comprehensive package of treatment and care services to IDUs are essential to expand treatment coverage and derive both individual and public health benefits of ART [47,48]. Our current study along with emerging work, including studies from the Eastern European region, further confirm that optimal outcomes can be achieved in all patients [49–51].
